# Outcomes of total hip replacement in adults with septic arthritis of the native hip joint: A systematic review

**DOI:** 10.1186/s42836-024-00292-w

**Published:** 2025-02-06

**Authors:** Teddy Cheong, Surya Varma Selvakumar, Ryan Kwang Jin Goh, Ing How Moo

**Affiliations:** 1 Department of Orthopaedic Surgery, Changi General Hospital, Singapore, 529889 Singapore; 2 Lee Kong Chian School of Medicine, Nanyang Technological University, Singapore, 308232 Singapore

**Keywords:** Hip Arthroplasty, Native, Septic arthritis, Outcomes

## Abstract

**Background:**

Septic arthritis is a debilitating condition that results in joint destruction and irreversible loss of joint function. Surgical treatment options include arthroscopy, resection arthroplasty, and total hip replacement (THR). Current literature on the treatment of septic arthritis of various joints includes periprosthetic or native joints. However, to our knowledge, a consolidated review that focuses solely on THR outcomes in a previously infected native hip is still lacking. This systematic review, for the first time, examined the clinical outcomes of THR, specifically in adults with septic arthritis of the native hip joint.

**Methods:**

PubMed, Embase, Cumulative Index to Nursing and Allied Health Literature (CINAHL), SCOPUS, Cochrane Library, grey literature, and bibliographic references were searched from inception to October 2023. Only case series or cohort studies published within the last 20 years assessing the outcomes of THR for native hip septic arthritis were included. Literature retrieval and data extraction were conducted by three independent reviewers. Re-infection rate and various functional outcomes, measured in terms of the Harris Hip Score (HHS), Visual Analogue Scale (VAS), Merle D'Aubigne and Postel (MAP), Western Ontario, McMaster Universities Arthritis Index (WOMAC) Short Form 12-Item (SF-12) scores, were analyzed. Range of motion (ROM) and limb length discrepancy (LLD) were also examined.

**Results:**

Against the relevant criteria, seven studies (six case series, one cohort study) involving 1243 patients were included. The patients aged from 18 to 78 years old. The reinfection rate ranged from 0 to 22.8%, with a mean rate of 19.6%. With regards to functional outcomes, the mean increase in HHS was from 39.5 to 48.92 and the increase in MAP ranged from 7.3 to 10.9. Improvement in LLD ranged from 2.28 to 3.52 cm, with all studies reporting < 1 cm of LLD postoperatively.

**Conclusion:**

THR, both single and two-staged, is an effective treatment option for septic arthritis of the native hip joint that and yields good functional outcomes and acceptable reinfection rates. However, more prospective and randomized trials are needed to establish clear protocols on antibiotic regimes, clinical criteria clearance, and optimal time from infection to joint replacement.

## Introduction

Septic arthritis is an orthopedic emergency that can result in rapid destruction of the joint and irreversible loss of joint function [1]. It is a debilitating condition with an estimated associated mortality rate of 11%. [2] A patient may develop septic arthritis via different modes of infection such as direct introduction, hematogenous seeding, or from a contiguous focus of infection. The synovial membrane of joints is well-vascularized with no basement plate, thus allowing easy hematological entry of bacteria into the joint. Bacteria colonize the joint, resulting in rapid proliferation and subsequent joint destruction via inflammatory processes and bacterial toxins. Early diagnosis with prompt drainage and appropriate antibiotics is key to avoiding joint destruction [3–6]. Hips with preserved anatomic structures can be treated with open or arthroscopic joint washout/debridement. [7] However, the treatment of destructive and recalcitrant septic hip arthritis can be complicated and remains controversial. In such cases, patients have joint destruction with osteomyelitis of the acetabulum and proximal femur, which may be treated with resection arthroplasty or total hip replacement (THR). [8] Girdlestone first described resection arthroplasty as a surgical option for the treatment of hip osteoarthritis, which involved resection of the proximal femur and debridement of soft tissue. [9] Although this option achieved good infection control, variable and poor functional outcomes were reported. [10, 11] THR is effective in treating hip osteoarthropathy [12]. This can be performed either as a single or two-staged surgery and the decision is influenced by the type of infection, such as quiescent or active hip infection. Active infection is defined as the presence of clinical and laboratory findings of local infection while a quiescent infection refers to a history of septic arthritis with no signs of active infection [13]. To our knowledge, there is no consolidated review that focuses solely on outcomes of THR in the setting of native hip septic arthritis. Therefore, this systematic review aimed to be the first to evaluate the reinfection rate and outcomes of THR in the management of septic native hip arthritis in adult patients.

## Methodology

This systematic review was conducted in accordance with the relevant requirements of the Preferred Reporting Items for Systematic Reviews and Meta-Analyses (PRISMA) Statement.

### Literature search strategy

To retrieve relevant literature for reviewing the clinical outcomes of THR in native hip septic arthritis, different databases were searched: MEDLINE (PubMed), Embase, Cumulative Index to Nursing and Allied Health (CINAHL), Cochrane Library, SCOPUS, grey literature (conference proceedings, industry white papers, Google Scholar) and bibliographic references were hand-searched to identify relevant studies. These databases were reviewed with the terms: (Hip) [MeSH] AND Septic Arthritis AND (Arthroplasty [MeSH] OR Replacement). The search was limited to articles published in the past 20 years until October 2023. Studies above case reports were included and systematic reviews were excluded. The search terms that were used are presented in Appendix Table 6.

### Inclusion and exclusion criteria

The inclusion criteria were as follows: (1) Type of Study: Published cohort studies, both retrospective and prospective, with an exclusive focus on the hip joint, published in the last 20 years, (2) Research Subjects: Patients > 18 years old regardless of gender, native hip joint septic arthritis, bacterial joint infection, (3) Intervention: THR, (4) Outcomes: Re-infection, functional outcomes, complications, (5) Studies written in English. The exclusion criteria included: (1) Type of Study: Case reports, systematic reviews and meta-analysis, studies including non-hip joints, (2) Research Subjects: Paediatric cases, periprosthetic joint infection cases, nonbacterial or tuberculosis (TB) septic arthritis, (3) Intervention: Studies in which THR was not reported on, (4) Outcomes: Studies with incomplete data, (5) Studies not written in English, (6) Studies containing unextractable data.

### Study selection and data extraction

The literature retrieval was conducted under the guidelines of established inclusion and exclusion criteria. Two reviewers, RG and SV, performed data extraction independently before compilation and cross-referencing. A third reviewer, TC, assisted in the cross-referencing process independently to minimize judgment errors and resolve conflicts. All three reviewers have prior experience in medical publications. The quantitative data extracted in this study included first author, publication year, sample size, intervention measures, outcomes and demographic details.

### Quality assessment of included studies

The overall methodological quality of the included studies was assessed using the methodological index for non-randomized studies (MINORS) tool, which utilizes a set of 12 criteria. The MINORS tool is simple, reliable, and has been widely used to assess the quality of non-randomized studies. It is comprehensive and has criteria that assess both comparative and non-comparative studies. The first eight criteria are specifically for non-comparative studies. The global ideal score for non-comparative and comparative studies is 16 and 24 respectively [14].

## Results

### Literature screening and results

**Fig. 1:**
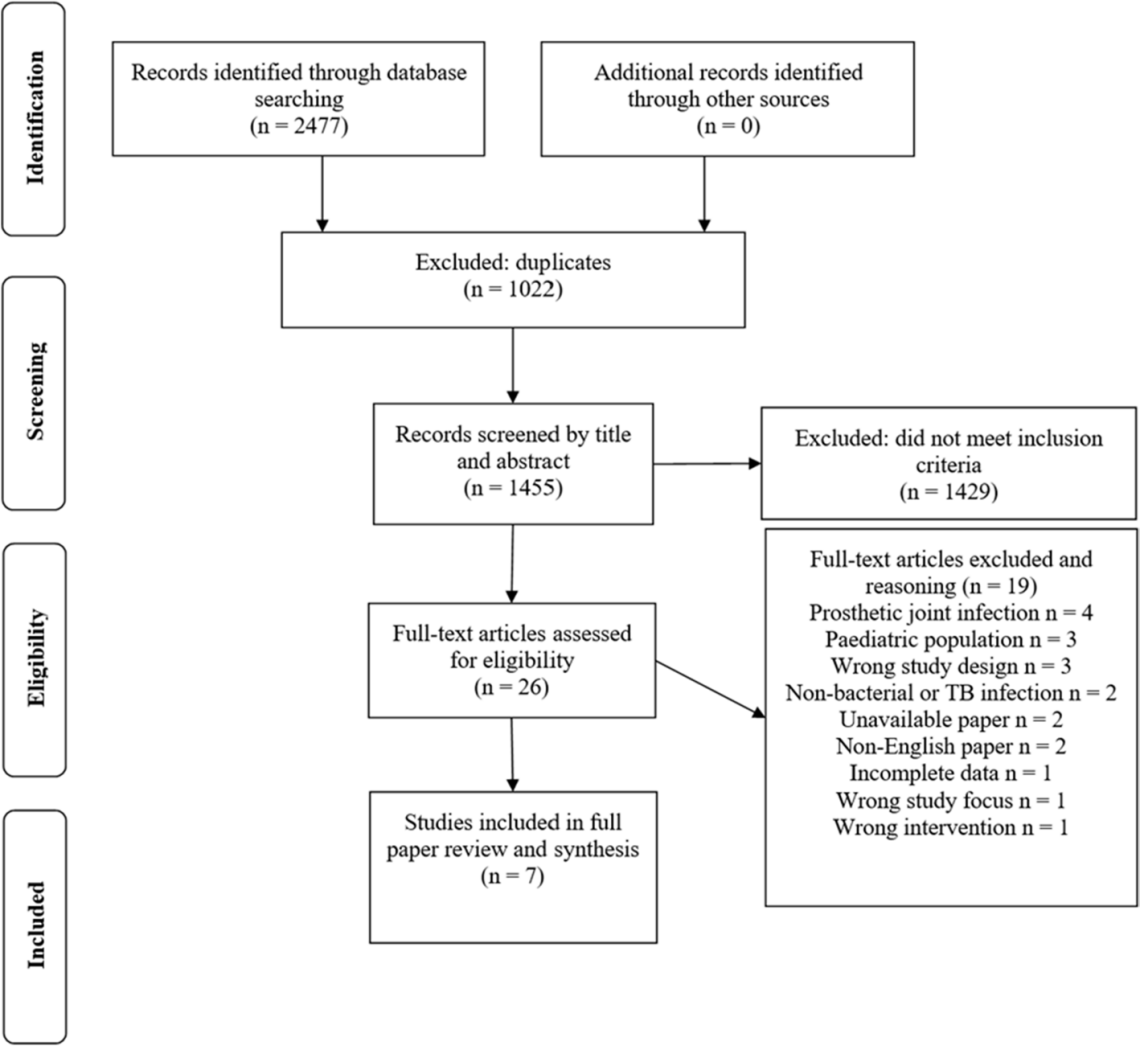
PRISMA flowchart of the search process

A total of 2477 studies were retrieved for initial evaluation, of which 1455 original articles remained after the duplicates were removed. Following the implementation of inclusion and exclusion criteria, the final sample size of ethically approved studies was narrowed down to seven (Fig. 1).

### Study characteristics

The included studies consisted of six case series and one cohort study. All included studies were of retrospective nature and in English. The total sample size was 1243. The age of patients ranged from 18 to 78 years old. The reinfection rate was reported in all studies. Functional outcomes reported included the Harrison Hip Score (HHS), Limb Length Discrepancy (LLD), Merle D’Aubigne and Postel (MAP), Visual Analogue Score (VAS), Western Ontario and McMaster (WOMAC), Short Form 12-Item (SF-12) and Range of Motion (ROM) (Table 1).

**Table 1:** Overview of included studies

First Author, Year	Study Design	Population Size (*n* = 1243)	Age	Outcomes Reported
Chen, 2008 [15]	Case Series	28	53 ± 26	Reinfection Rate, HHS, LLD
Dubin 2022 [16]	Retrospective Cohort Study	1030	60 ± 3	Reinfection Rate
Huang, 2010 [17]	Case Series	14	54.3 ± 25.3	Reinfection Rate, MAP
Poignard, 2011 [18]	Case Series	20	25 ± 18	Reinfection Rate, VAS
Ye, 2023 [19]	Case Series	90	Male: 45.64 ± 12.15; Female: 66.29 ± 1.66	Reinfection Rate, HHS, VAS, WOMAC, LLD, ROM
Anagnostakos, 2016 [20]	Case Series	16	59.7 ± 27.7	Reinfection Rate
Zeng, 2019 [21]	Case Series	45	45.9 ± 22.1	Reinfection Rate, HHS, VAS, WOMAC, MAP, SF-12, LLD, ROM

HHS, Harrison Hip Score; LLD, Limb Length Discrepancy; MAP, Merle D’Aubigne and Postel; VAS, Visual Analogue Score; WOMAC, Western Ontario and McMaster; SF-12, Short Form 12-Item; ROM, Range of Motion

### Treatment details

Treatment characteristics are described in Table 2. The antibiotic duration varied across the studies in this review, ranging from days to months. All studies except the study by Dubin et al. reported on their antibiotic regimes. For active septic arthritis, there are many guidelines on the recommended antibiotic duration, but at least 4 weeks of therapy appears to be the generally accepted duration [22–24]. Studies involving two-staged THR for active infection reported an antibiotic duration of ≥ 4 weeks except the study by Huang et al*.* [15, 17, 18, 20]. With regards to single-stage THR for quiescent infection, studies reported relatively short antibiotic durations, with Zeng’s study having the shortest duration of intraoperative followed by two days of antibiotics [19, 21].

**Table 2:** Treatment characteristics

First author, year	Nature of septic arthritis	Type of THR	Time to THR (Single-stage: time from infection; Two-stage: interval from 1st to 2nd stage)	Pre-THR antibiotics	Post-THR antibiotics	Antibiotics utilised	Antibiotics duration of ≥ 4 weeks?
Chen, 2008 [15]	Active septic arthritis	Two-stage	3.64 months (range: 1–7)	Pre-1st stage: 4–6 weeks; Pre-2nd stage: 4 weeks (range: 2–16)	Most cases: Until negative postoperative c/s result; 3 cases with positive postoperative c/s: 6 weeks post-THR	Empirical oxacillin and gentamycin followed by culture-directed	Yes
Dubin 2022 [16]	Quiescent septic arthritis	Single-stage	0–6 months for 859 cases; 6–12 months for 193 cases	Not specified	Not specified	-	-
Huang, 2010 [17]	Active septic arthritis	Two-stage	3 months (range: 1–8)	Pre-1st stage: duration not specified; Pre-2nd stage: IV 1-week culture-directed	IV 3 days	Empirical 1st-generation cephalosporin and gentamycin followed by culture-directed	No
Poignard, 2011 [18]	Active septic arthritis	Two-stage	12 months (range: 6–12)	Varied regime: Most cases received 12 to 26 weeks	12 weeks	Empirical choice not stated; Culture-directed	Yes
Ye, 2023 [19]	Quiescent septic arthritis	Single-stage	Not specified	IV 30 min pre-operation	IV 5–7 days	2nd-generation cephalosporin	No
Anagnostakos, 2016 [20]	Active septic arthritis	Two-stage	3 months (range: 1–9)	4 weeks IV + 2 weeks oral	None	Not specified	Yes
Zeng, 2019 [21]	Quiescent septic arthritis	Single-stage	34.2 years (range: 13–65)	Intraoperative IV	IV 2 days	Norvancomycin	No

THR, Total Hip Replacement; IV, Intravenous; c/s, Culture

### Reinfection rate

The rate of re-infection was reported in all studies, ranging from 0% to 22.8%, with Dubin’s study having the highest rate of reinfection. The overall mean reinfection rate was 19.6%. The mean reinfection rate in single-stage THR and two-stage THR was 20.2% and 11.5% respectively (Table 3).

**Table 3:** Reinfection rate

First author, year	Reinfection rate, THR type
Huang, 2010 [17]	0% (0/14), Two-stage
Chen, 2008 [15]	14.3% (4/28), Two-stage
Poignard, 2011 [18]	15% (3/20), Two-stage
Anagnostakos, 2016 [20]	12.5% (2/16), Two-stage
Ye, 2023 [19]	0% (0/90), Single-stage
Zeng, 2019 [21]	0% (0/45), Single-stage
Dubin, 2022 [16]	22.8% (235/1030), Single-stage
Mean Reinfection Rate	➢Overall: 19.6% (244/1243) ➢Two stage THR: 11.5% (9/78) ➢Single-stage THR: 20.2% (235/1165)

THR, Total Hip Replacement

### Functional outcomes

Functional outcome scores reported included HHS, MAP, VAS, WOMAC, and SF-12. LLD and ROM were also described in several studies. Scores used or reported by less than three studies, such as the WOMAC, VAS, SF-12, and ROM, were excluded from the main text to avoid heterogeneity but are included in Appendix Table 7 under the appendix section for completion in coverage of the extracted data. HHS, MAP, and LLD are included in the main text as they each had three studies reporting on these outcomes.

**Fig. 2:**
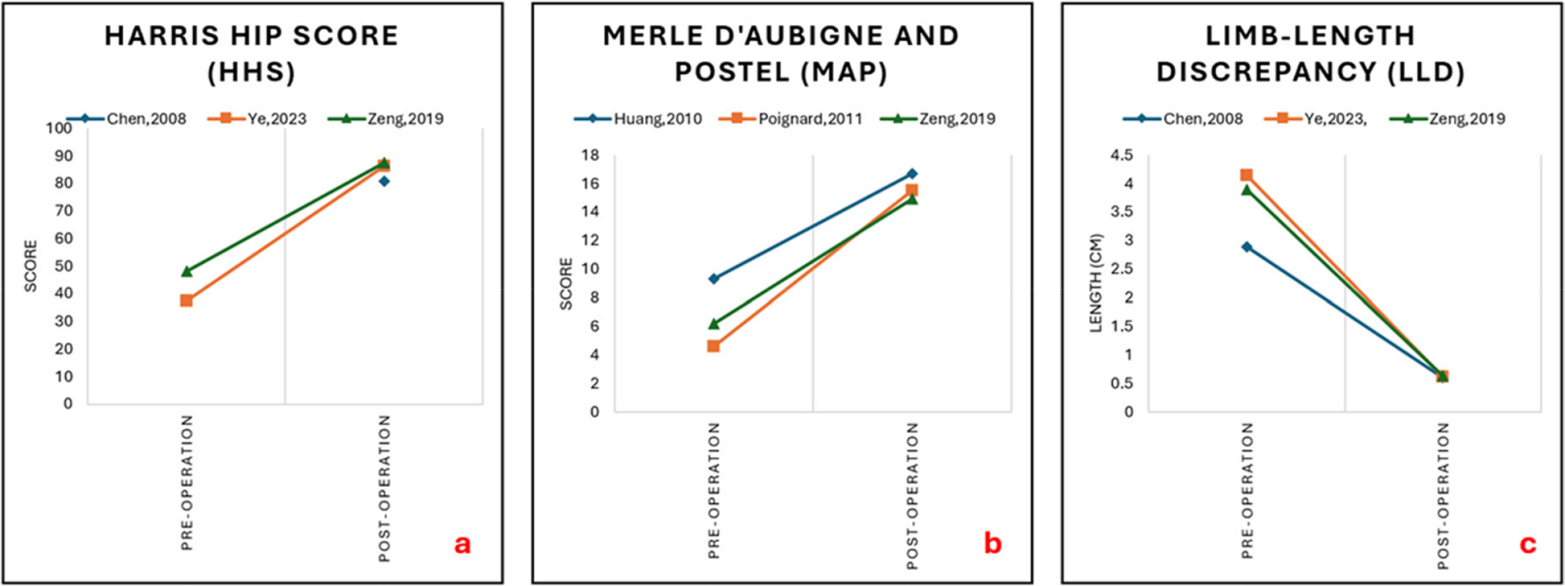
Improvement in functional outcome scores across the studies (2a: HHS, 2b: MAP, 2c: LLD)

The mean postoperative HHS score ranged from 80.9 to 87.6, with improvement in scores ranging from 39.5 to 48.92 [15, 19, 21]. The mean postoperative MAP score ranged from 14.9 to 16.7 with improvement in scores ranging from 7.3 to 10.9 [17, 18, 21]. All studies had < 1 cm LLD postoperatively, with improvement in LLD ranging from 2.28 to 3.52 cm [15, 19, 21] (Table 4). Figure 2 illustrates the improvement in these outcomes.

**Table 4:** Common functional outcomes reported

		**Chen, 2008 **[15]	**Ye, 2023 **[19]	**Zeng, 2019 **[21]	**Huang, 2010 **[17]	**Poignard, 2011 **[18]
**Type of THR**		Two-stage	Single-stage	Single-stage	Two-stage	Two-stage
**HHS**	Preoperation	-	37.34 ± 11.25	48.1 ± 10.3 (range, 32–65)	-	-
	Postoperation	80.9 (range, 48–97)	86.26 ± 10.64	87.6 ± 7 (range, 77–98)	-	-
	Mean Change	-	+ 48.92	+ 39.50	-	-
**LLD**	Preoperation	2.89 cm (range: 2–4 cm)	4.14 cm ± 1.18	3.89 cm ± 1.23	-	-
	Post-operation	0.61 cm (range: 0–2.5 cm)	0.62 cm ± 0.13	0.64 cm ± 0.62	-	-
	Mean Change	− 2.28	− 3.52	− 3.25	-	-
**MAP**	Preoperation	-	-	6.2 ± 1.4 (range, 3–9)	9.3 (range, 5–15)	4.6
	Postoperation	-	-	14.9 ± 1.1 (range, 12–18)	16.7 (range, 15–18)	15.5
	Mean Change	-	-	+ 8.70	+ 7.30	+ 10.90

THR, Total Hip Replacement; HHS, Harrison Hip Score; LLD, Limb Length Discrepancy; MAP, Merle D’Aubigne and Postel

### Quality assessment of included studies

The overall methodological quality of the included studies was assessed using the MINORS tool. Only study by Dubin et al. was comparative in nature [16] (Table 5).

**Table 5:** Methodological index for non-randomized studies tool assessment of the seven studies

**Criteria**	**Chen 2008 **[15]	**Dubin 2022 **[16]	**Huang 2010 **[17]	**Poignard 2011 **[18]	**Ye 2023 **[19]	**Anagnostakos 2016 **[20]	**Zeng 2019 **[21]
A clearly stated aim	1	2	1	2	1	1	2
Inclusion of consecutive samples	2	2	1	1	2	0	0
Prospective collection of data	2	2	2	2	2	2	2
Endpoints appropriate to aim of study	2	2	2	1	2	1	2
Unbiased assessment of study endpoint	0	0	0	0	0	0	0
Aim-congruent follow-up period	2	2	1	2	0	1	2
Loss of samples (< 5%)	2	2	2	2	1	1	1
Prospective calculation of study size	0	1	0	0	0	0	0
Adequate control group	NA	2	NA	NA	NA	NA	NA
Contemporary groups	NA	0	NA	NA	NA	NA	NA
Baseline equivalence of groups	NA	2	NA	NA	NA	NA	NA
Adequate statistical analyses	NA	2	NA	NA	NA	NA	NA
Total	11/16	19/24	9/16	10/16	8/16	6/16	9/16

## Discussion

Our review demonstrated that THR for patients with destructive native septic hip arthritis can achieve good clinical outcomes regardless of the type of infection (quiescent or active). This was evident in the improvements in terms of functional outcome scores, such as the HHS and MAP, and clinical parameters like LLD. This finding is in keeping with existing literature on the functional outcomes following a THR in a septic hip [25–27]. The HHS is a commonly used scoring system for the hip and in this review. The mean improvement ranged from 39.5 to 48.92, which is comparable with what was seen in THR performed for cases of osteoarthritis where the mean improvement in the score ranged from 37 to 43 [28–30]. LLD is an important clinical outcome of THR, and it has been shown that LLD of > 1 cm can lead to poor results [31]. In this review, the preoperative LLD ranged from 2.89 to 3.89 cm and the postoperative LLD was < 1 cm throughout the studies, suggesting that THR in native hip septic arthritis can achieve equal leg length.

The mean overall rate of reinfection was 19.6% in this review. This is comparable with the existing results of previous studies, which reported that the reinfection rate after THR in hip septic arthritis or prosthetic joint infection (PJI), could range from 0 to 27% (25, 26, 32–34). The studies on quiescent infection showed that the mean reinfection rate after THR was relatively high (20.2%). This was mainly because of Dubin’s study, which had a reinfection rate of 22.8%, whereas Zeng and Ye reported a reinfection rate of 0% [16, 19, 21]. Possibly Dubin’s high rate of infection could have been ascribed to the presence of risk factors for PJI amongst his population. For instance, obesity has been shown to increase the risk of PJI [35] and 53.1% of Dublin’s population had obesity, the rate being much higher than that of the other studies included in this review.

There is no clear evidence on the ideal post-infection duration in which an arthroplasty should be performed. This includes the setting of native hip septic arthritis. A study by Bettencourt et al. found that THR within five years of septic arthritis had a higher risk of infection [36]. International Consensus had an 87% agreement that arthroplasty should be performed no earlier than three months from infection. However, they found no concrete evidence to support this [37]. In contrast, a study by Tan et al. reported no difference in THR PJI rate between replacement within five months vs. after five months of infection [38]. In this review, among the two-stage THR studies regarding active infection, Anagnostakos had the shortest duration of 3 months between the 1st and 2nd stage operations and had no cases of reinfection, whereas Poignard had the longest duration of 12 months but still reported a 15% reinfection rate [18, 20]. However, Poignard’s study consisted of patients with sickle cell anemia and other comorbidities. His treatment approach was also different in that joint aspiration and antibiotic therapy went first and only patients with joint destruction at follow-up or recurrent septic dislocations underwent arthrotomy and THR. These may have contributed to the high reinfection rate seen in his study [18]. With regards to single-stage THR, Dubin et al. reported that patients with septic hip arthritis who underwent THR within six months of infection were at higher risk of developing PJI than those who underwent THR within 6–12 months of infection [16].

Moreover, no consensus has been reached on the clinical criteria used to determine the optimal condition for a THR. In this review, there were a wide variety of criteria used among the included studies, ranging from pure laboratory-based [19] to different combinations of clinical parameters and infection markers [15, 17, 20]. The heterogeneity suggests that a universally-accepted criterion is lacking to determine the optimal condition for THR for native hip septic arthritis. More randomized trials are needed to arrive at a consensus on both the optimal time and condition for a THR to be performed. Our recommendation of treatment would be that arthrotomy and debridement of all necrotic and infected tissues should be performed first, followed by appropriate antibiotic therapy. In two-stage THRs for active infection, it is safe to perform the 2nd stage THR 3 months after the 1st stage operation, given the infection eradication was microbiologically (e.g., aspiration), clinically, and radiologically confirmed. For quiescent native hip septic arthritis, THR should be performed only after 1 year.

The principle of treatment of septic arthritis is joint drainage in combination with appropriate antibiotics [3]. The studies involving quiescent infection had relatively short antibiotic durations, with only perioperative/intraoperative doses and a few days of postoperative antibiotics. There were no courses of pre-THR antibiotics given in the single-stage THRs, yet they still had a reinfection rate of 0% [15, 17]. These results suggest that prolonged antibiotic use is not needed in THR for quiescent septic arthritis of the native hip.

Multiple guidelines are available recommending antibiotic duration in active septic arthritis. The European Bone and Joint Society (EBJIS) recommends 1–2 weeks of IV therapy followed by 2–3 weeks of oral antibiotics [22]. In France, experts from various disciplines recommended a total of 4–6 weeks of antibiotics (IV then oral) [23] and in Korea, the recommendation was a total of 4–6 weeks of antibiotics, with IV therapy lasting for at least two weeks [24]. Based on these guidelines, it appears that at least four weeks of antibiotic therapy is the general advice for active septic arthritis. In 2023, Joo et al. conducted a retrospective study on optimal antibiotic therapy for patients with native joint septic arthritis and found that four weeks or less of antibiotics was a risk factor for re-infection [39]. For the studies involving active infection in this review, only Huang had < 4 weeks of antibiotics usage. Despite this, his study had 0% reinfection [17]. However, Anagnostakos had six weeks of antibiotics and still had cases of reinfection (12.5%) [20]. Poignard et al. had the longest total antibiotic regime, including 12 weeks post-THR, but still reported a 15% re-infection rate. However, as mentioned earlier, Poignard’s study concerned sickle cell anemia patients with various comorbidities and this could affect infection eradication, leading to a higher reinfection rate despite the longer antibiotic regime. The variation in results in this study may cast doubts over the efficacy of protracted antibiotics and their ideal duration in the setting of THR for active native septic hip arthritis. The wide variety of antibiotic regimes used in the included studies suggests that there is no standard protocol for antibiotic use. This area warrants more research before a conclusion is reached on the optimal antibiotic regime in patients who receive THR for native septic hip arthritis. Our view is that a perioperative dose of antibiotics as per a primary THR for quiescent infection is adequate and 3 days of IV antibiotics after the 2nd stage THR for active septic arthritis.

### Limitations

Our study has several limitations. Firstly, the number of studies included is low due to the inclusion and exclusion criteria. This is likely due to the fact that limited number of studies focused on THR in solely native hip septic arthritis in adults. Secondly, no single functional outcome score was used across all the included studies, which made it difficult to directly compare each study’s functional outcomes. Lastly, the lack of standardization between the papers on the reporting of antibiotic regimes, surgical techniques, and patient selection likely contributed to the heterogeneity across the studies, which may limit the power of the results as these factors undoubtedly affect outcomes. Overall, not a clear consensus was reached on issues such as antibiotic regime, clinical criteria for clearance to perform THR, and the optimal time between infection and arthroplasty.

## Conclusion

THR, both single and two-stage, is an effective treatment option for septic arthritis of the native hip joint that produces good functional outcomes and acceptable reinfection rates. However, more prospective and randomized trials are needed to establish clear protocols on the antibiotic regime, clinical criteria clearance, and optimal time from infection to joint replacement.

## Data Availability

All data generated or analyzed during this study are included in this published article.

## Appendix

**Table 6:** Database and search terms

**Database**	**Search Terms**
MEDLINE (PubMed) Embase Cumulative Index to Nursing and Allied Health (CINAHL) Cochrane Library SCOPUS	(Hip) [MeSH] AND Septic Arthritis AND (Arthroplasty [MeSH] OR Replacement)

**Table 7:** Other functional outcomes reported

		**Ye, 2023 [15]**	**Zeng, 2019 [17]**
**WOMAC**	Pre-operation	28.78 ± 5.32	67.6 ± 4.7 (range, 61–75)
	Post-operation	80.62 ± 13.66	40.8 ± 11.6 (range, 22–66)
	Mean Change	+51.84	−26.80
**VAS**	Pre-operation	8.08 ± 2.12	^a^None: 39 ^b^Mild: 3 ^c^Moderate: 2 ^d^Severe: 1
	Post-operation	4.32 ± 1.16	None: 42 Mild: 2 Moderate: 0 Severe: 0
	Mean Change	–3.76	None: +3, Mild: –1, Moderate: –2, Severe: –1
**SF-12**	Pre-operation	-	10.8 ± 3.8 (range, 6–16)
	Post-operation	-	20.7 ± 2.2 (range, 18–24)
	Mean Change	-	+9.90
**ROM**	Pre-operation	53.76 ± 12.37	-
	Post-operation	128.24 ± 10.53	-
	Mean Change	+74.48	-

^a^None (0 points), ^b^Mild (1-3 points), ^c^ Moderate (4-6 points), ^d^ Severe (7-10 points); VAS, Visual Analogue Score; WOMAC, Western Ontario and McMaster; SF-12, Short Form 12-Item; ROM, Range of Motion

WOMAC and VAS showed improvement in two studies [15, 17] ROM and SF-12 were described in only one paper and showed improvement [15, 17].

## Abbreviations

THR: Total Hip Replacement
HHS: Harris Hip Score
VAS: Visual Analogue Scale
MAP: Merle D’Aubigne and Postel
WOMAC: Western Ontario and McMaster Universities Arthritis Index
SF-12: Short Form 12-Item
ROM: Range of motion
LLD: Limb length discrepancy
TB: Tuberculosis
PRISMA: Preferred Reporting Items for Systematic Reviews and Meta-Analyses
CINAHL: Cumulative Index to Nursing and Allied Health
MINORS: Methodological index for non-randomized studies
PJI: Prosthetic joint infection
CRP: C-reactive protein
ESR: Erythrocyte sedimentation rate
EBJIS: European Bone and Joint Society
IV: Intravenous
